# A randomized controlled trial of an educational video to improve quality of bowel preparation for colonoscopy

**DOI:** 10.1186/s12876-016-0476-6

**Published:** 2016-06-17

**Authors:** Jin-Seok Park, Min Su Kim, HyungKil Kim, Shin Il Kim, Chun Ho Shin, Hyun Jung Lee, Won Seop Lee, Soyoung Moon

**Affiliations:** Digestive Disease Center, Department of Internal Medicine, Inha University School of Medicine, 27 Inhang-ro, Jung-gu, Incheon, 400-711 South Korea

**Keywords:** Bowel preparation, Colonoscopy, Video

## Abstract

**Background:**

High-quality bowel preparation is necessary for colonoscopy. A few studies have been conducted to investigate improvement in bowel preparation quality through patient education. However, the effect of patient education on bowel preparation has not been well studied.

**Methods:**

A randomized and prospective study was conducted. All patients received regular instruction for bowel preparation during a pre-colonoscopy visit. Those scheduled for colonoscopy were randomly assigned to view an educational video instruction (video group) on the day before the colonoscopy, or to a non-video (control) group. Qualities of bowel preparation using the Ottawa Bowel Preparation Quality scale (Ottawa score) were compared between the video and non-video groups. In addition, factors associated with poor bowel preparation were investigated.

**Result:**

A total of 502 patients were randomized, 250 to the video group and 252 to the non-video group. The video group exhibited better bowel preparation (mean Ottawa total score: 3.03 ± 1.9) than the non-video group (4.21 ± 1.9; *P* < 0.001) and had good bowel preparation for colonoscopy (total Ottawa score <6: 91.6 % vs. 78.5 %; *P* < 0.001). Multivariate analysis revealed that males (odds ratio [OR] = 1.95, *P* = 0.029), diabetes mellitus patients (OR = 2.79, *P* = 0.021), and non-use of visual aids (OR = 3.09, *P* < 0.001) were associated with poor bowel preparation. In the comparison of the colonoscopic outcomes between groups, the polyp detection rate was not significantly different between video group and non-video group (48/250, 19.2 % vs. 48/252, 19.0 %; *P* = 0.963), but insertion time was significantly short in video group (5.5 ± 3.2 min) than non-video group (6.1 ± 3.7 min; *P* = 0.043).

**Conclusion:**

The addition of an educational video could improve the quality of bowel preparation in comparison with standard preparation method.

**Trial registration:**

Clinical Research Information Service KCT0001836. The date of registration: March, 08^th^, 2016, Retrospectively registered.

**Electronic supplementary material:**

The online version of this article (doi:10.1186/s12876-016-0476-6) contains supplementary material, which is available to authorized users.

## Background

Colonoscopy is an important modality for diagnosing and preventing colorectal cancer [1]. Adequate bowel cleansing is a key factor for complete visualization of the colonic mucosa in colonoscopy, which can increase polyp detection and reduce adverse events such as perforation [2, 3]. In contrast, inadequate bowel preparation reduces procedure quality, induces difficult and time-consuming procedures, and increases the need for repeat examinations scheduled at earlier intervals [3, 4]. However, bowel preparation is typically inadequate in an estimated 10–20 % of patients undergoing colonoscopy [3, 5], and 12–22 % of excess colonoscopy-related costs are due to suboptimal bowel preparation [6]. Although many reasons contribute to unsatisfactory bowel cleansing, such as inpatient status, chronic constipation, tricyclic antidepressants, male sex, and later colonoscopy start time, patient compliance could play a major role in poor bowel preparation. This may occur by means of an insufficient amount of preparation consumption, improper start time of preparation, incorrect length of preparation time, and use of antidepressants or narcotics [7, 8]. Thus, the education of patients before colonoscopy is very important to ensure compliance.

Patient education programs are used in many gastroenterology units to prepare patients for colonoscopy, and a few studies have reported that patient education led to improvements in the quality of colonoscopy preparation, by enhancing patient compliance [9, 10]. In other literature, however, the use of patient education for bowel preparation failed to demonstrate any effect in quality of bowel preparation [11, 12]. Evidence supporting the efficacy of bowel preparation education is inconsistent and has not been fully evaluated.

We designed a simple bowel preparation video for patients about to undergo colonoscopy with an emphasis on the importance of adhering to instructions for proper bowel preparation. The video consisted of pictorial demonstrations, subtitles, and simplified instructions. The aim of this study was to measure the effect of this simple video on the quality of bowel preparation during colonoscopy and its impact on clinically relevant outcomes, such as polyp detection. In addition, we investigated factors associated with poor bowel preparation.

## Methods

### Patients

This study was conducted from October 2014 to February 2015. We enrolled outpatients over 18 years of age undergoing a screening colonoscopy at the endoscopy center of Inha University Hospital. Patients with inflammatory bowel disease, colorectal malignancy, or infectious colitis, prior colonic resection, non-polyethylene glycol solution-based bowel preparation, and/or a family history of colorectal neoplasia were excluded. Patients who had insufficient intellectual capacity to understand the survey process were also excluded. All enrolled patients visited at the endoscopy center and provided written informed consent after receiving explanations about colonoscopy by the endoscopy center staff. Patients were randomized to either the video group or the control group by a random-number generator at the time of colonoscopy scheduling.

### Bowel preparation

All patients received regular instructions at the time of their appointment to discuss the colonoscopy. One gastrointestinal staff provided education about colonoscopy, including the exact preparation instructions and information on the importance of bowel preparation and the adverse effects of the agents used. Patients also received a preparation manual with clear written instructions. Patients were prescribed polyethylene glycol electrolyte powder (Coolprep powder sachets containing 50 g polyethylene glycol 3350, 1.35 g sodium chloride, 3.75 g sodium sulfate, 0.51 g potassium chloride, ascorbic acid 2.35 g, and sodium ascorbate 2.95 g; Taejoon Pharmaceutical Company, Korea) for bowel preparation. All patients were instructed to end their regular meal before 18:00 and allowed to drink only clear water after regular meal. They were asked to drink two sachets of Coolprep powder dissolved in one liter of water within 60 min at 20:00–21:00 the day before the colonoscopy, as a bowel purgative. They were asked to drink the solution again in the same way at 06:00–07:00 on the day of colonoscopy. Patients were encouraged to drink more clear liquids after purgatives for adequate hydration before colonoscopy. All enrolled patients were scheduled for a morning session (before 13:00).

### Design of the education video

**Fig. 1:**
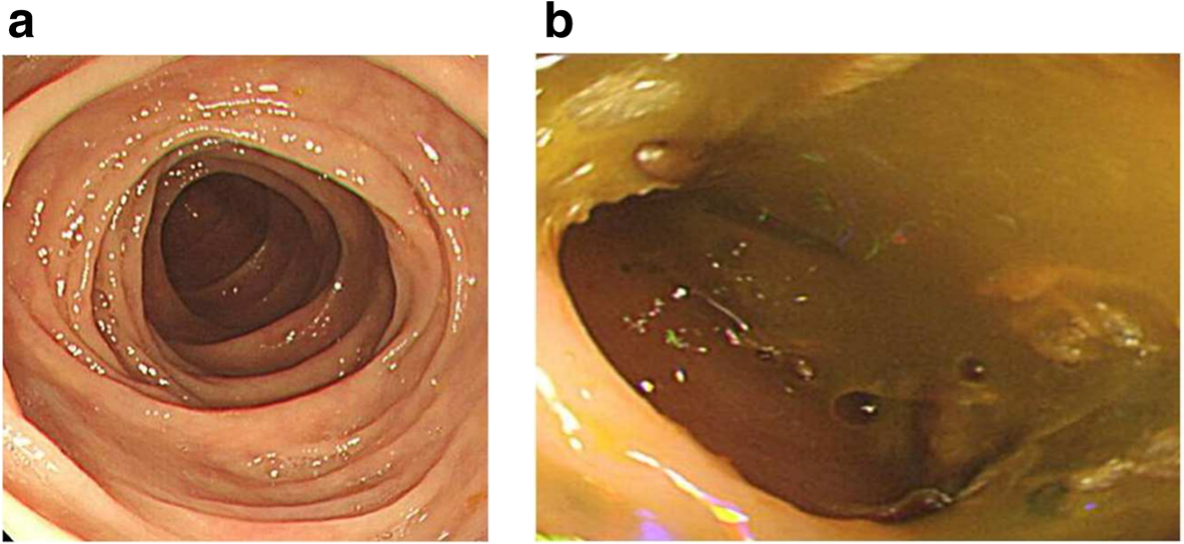
Distribution of patients with screening colonoscopy between the study groups

We designed a 6-min bowel preparation video, which included instructions with pictures, video, and subtitles to supplement the standard written preparation instructions. It also included photographs of optimal and poor preparation for patients to visually understand the clinical significance of bowel preparation (Fig. 1). The educational video was uploaded to a website. Only the patients allocated to the video group were provided with the website address. They were encouraged to watch the video on the day before the colonoscopy (Additional file 1: Video 1).

### Study design

The study followed a prospective, randomized controlled design. The colonoscopy-performing physician and members of the research team were blinded to the allocation. On the day of colonoscopy, all enrolled patients were asked to complete a three-page multiple choice questionnaire in Korean before the procedure. It included questions evaluating educational level, income level, demographic information (age and sex), previous colonoscopy history, and health behavior (smoking and alcohol). The questionnaire also confirmed whether a patient assigned to the video group watched the educational video. Endoscopists were instructed not to ask patients whether they had viewed the educational video. Colonoscopies were carried out by one of four endoscopists, each of whom had a minimum experience of 1000 colonoscopies and all of them were educated for a week by the typical images of the Ottawa Bowel Preparation Quality Scale (Ottawa score) before the starting study [13]. After the procedure, the endoscopist completed an assessment form evaluating the quality of the bowel preparation by using the Ottawa score. The Ottawa score requires the endoscopist to rate cleansing in three segments (right colon, mid-colon, and rectosigmoid colon) on a scale of 0 to 4 (with 0 indicating perfect cleansing) and an overall fluid quantity on a scale of 0 to 2 (with 0 indicating no fluid in the colon) [13]. The individual scores were summed to create a total score, with lower scores indicating better preparation [13]. Insertion time, withdrawal time, and colonoscopic findings for all patients were recorded also in the assessment form by the endoscopist.

### Ethics, consent and permissions'

The study protocol and amendment were approved by the Institutional Review Board of Inha university hospital (IUH-IRB 14-2262). The informed consent of the study was obtained from all patients. The methods of current study adhered to CONSORT guidelines.

### Definitions

Poor bowel preparation was defined by total Ottawa score ≥6 at the time of colonoscopy. The insertion time was defined as the interval between the start of the procedure and arrival at the cecum with identification of the appendiceal orifice. Withdrawal time was defined as the interval between withdrawal from the cecum and removal of the colonoscope from the patient.

### Outcome measurements

The primary outcome of this study was the quality of bowel preparation as assessed by the endoscopist using the Ottawa score. Secondary outcomes were colonoscope insertion and withdrawal time and the number of polyps and adenomas detected during colonoscopy. We also performed a secondary analysis of demographic information, education level, income, and any previous colonoscopies.

### Statistical analysis

A sample size was calculated to obtain a satisfactory estimation with a significance level (α) of 0.05, a power of 80 % with a two-tailed test, and an expected 10 % difference in the rate of colonic cleansing. The expected 10 % difference was based on a previous published study on the efficacy of education for bowel preparation [14]. In the results of the calculation, the sample size required per group was ≥189 patients. We anticipated that the dropout ratio would be 20 %. Therefore, we estimated a total of 500 patients would be required for this study.

On the baseline characteristics analysis, continuous data were calculated using Student’s *t*-test, and the chi-square test was used for categorical variables. The two groups’ bowel preparation scores were compared using the chi-square test. A logistic regression model was used to assess factors associated with poor bowel preparation (Ottawa score ≥6). The odd ratios (OR) and 95 % confidence intervals (95 % CI) were determined. A *P* value <0.05 was considered statistically significant. Statistical analysis was performed using SPSS (SPSS for Windows, version 19.0, SPSS Inc., Chicago, IL, USA).

## Results

### Patient characteristics

**Fig. 2:**
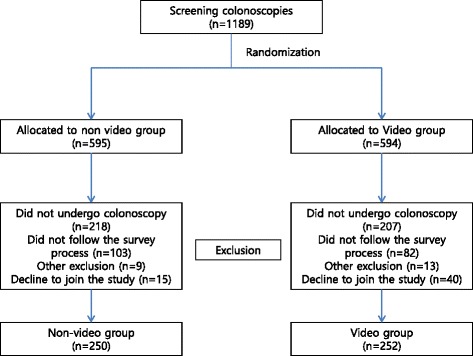
Pictures of adequate and inadequate bowel preparation as shown in the educational video

From October 2014 to February 2015, 1189 outpatients were considered for this study. Among them, 687 were excluded, 425 did not keep procedure appointment, 185 did not follow the survey process for bowel preparation including the 82 patients who not access the educational video, 22 met exclusion criteria and 55 declined to participate. Finally, a total of 502 patients were prospectively enrolled and randomized to the video group (*n* = 252) and the control group (*n* = 250) (Fig. 2). The mean age of patients in the control group was 47.3 ± 9.2 years, and in the experimental group was 49.2 ± 8.6 years (*P* = 0.017). Except the age, the two groups had similar baseline characteristics, including sex, body mass index, history of previous colonoscopy, health behaviors (alcohol, smoking), education level, economic level, and previous abdominal operation history (Table 1).

**Table 1:** Baseline characteristics of patients (*n* = 502)

Characteristic	Non-video group	Video group	*P* value
No. of patients	252	250	
Sex			0.416
Male (%)	167 (66.3 %)	157 (62.8 %)	
Female (%)	85 (33.7 %)	93 (37.2 %)	
Age, mean ± SD	47.3 ± 9.2	49.2 ± 8.6	0.017
BMI, Kg/m^2^	24.7 ± 3.4	24.3 ± 3.0	0.163
Previous colonoscopy (%)			0.269
Yes	143 (56.7)	154 (61.6)	
No	109 (43.3)	96 (38.4)	
Diabetes mellitus	13 (5.2)	22 (8.8)	0.118
Smoking History (%)			0.98
Non smoker	132 (53.8)	132 (53.0)	
Past smoker	46 (18.8)	48 (19.3)	
Current smoker	67 (27.4)	69 (27.7)	
Alcohol History (%)			0.169
None	71 (29.0)	92 (36.9)	
Moderate drinking	120 (49.0)	109 (43.8)	
Heavy drinking	54 (22)	48 (19.3)	
Abdominal operation (%)			0.839
Yes	34 (13.9)	33 (13.3)	
No	211 (86.1)	216 (86.7)	
Education level (%)			0.286
< Middle school	2 (0.8)	4 (1.6)	
Middle school	7 (2.9)	17 (6.8)	
High school	90 (36.7)	89 (35.7)	
Graduate	121 (49.4)	114 (45.8)	
Postgraduates	25 (10.2)	25 (10.1)	
Annual income level, $ (%)			0.486
< 10.000	3 (1.2)	7 (2.8)	
10,000–20,000	7 (2.9)	13 (5.2)	
> 20,000–30,000	30 (12.2)	29 (11.6)	
> 30,000–40,000	43 (17.6)	42 (16.9)	
> 40,000	162 (66.1)	158 (63.5)

### Outcomes of bowel preparation according to the Ottawa score

There was a statistically significant difference between the two groups with regard to quality of colonoscopy preparation. The mean Ottawa total scores differed significantly between the control and experimental group (mean Ottawa total score = 4.21 ± 1.9 versus 3.03 ± 1.9, *P* < 0.001). Using the individual components of the Ottawa score, the video group had better ratings for cleanliness for the right colon, mid colon, and recto-sigmoid. However, the fluid level was not significantly different (*P* = 0.203). Additionally, the proportion of patients who achieved adequate preparation (Ottawa total score ≤5) differed significantly between the non-video group (78.5 %) and the video group (91.6 %; *P* < 0.001) (Table 2).

**Table 2:** Ottawa Bowel Preparation Quality Scale

	Non-video group	Video group	
	(*n* = 252)	(*n* = 250)	*P* value
Segment (mean ± SD)			
Right colon	1.26 ± 0.8	0.93 ± 0.8	<0.001
Mid-colon	1.13 ± 0.6	0.76 ± 0.7	<0.001
Recto-sigmoid	1.14 ± 0.6	0.77 ± 0.6	<0.001
Fluid	0.68 ± 0.6	0.61 ± 0.62	0.203
Total scope	4.21 ± 1.9	3.03 ± 1.9	<0.001
Good bowel preparation for colonoscopy (OBPS <6), no, (%)	198 (78.5)	229 (91.6)	<0.001

*Abbreviations*: *OBPS* Ottawa Bowel Preparation Quality Scale

### Factors associated with poor bowel preparation

Logistic regression analyses were performed to identify any significant factors for poor bowel preparation. The factors analyzed were age, sex, body mass index, history of previous colonoscopy, history of abdominal surgery, diabetes mellitus, educational level, annual income level, and educational video. The univariate analysis indicated that male sex (OR = 2.14; *P* = 0.024) diabetes mellitus (OR = 3.01; *P* = 0.020), and no educational video viewing (OR = 3.06; *P* < 0.001) were factors significantly associated with poor bowel preparation for colonoscopy. The multivariate analysis revealed that male sex (OR = 1.95; *P* = 0.029), diabetes mellitus (OR = 2.79; *P* = 0.021), and no educational video viewing (OR = 3.09; *P* < 0.001) were factors also significantly associated with poor bowel preparation (Table 3).

**Table 3:** Logistic analysis of factors for a poor bowel preparation (Ottawa score ≥ 6)

	Univariate analysis		
Factor	OR	95 % CI	*P* value
Age	0.99	0.96–1.02	0.989
Sex			
Male	2.14	1.11–4.13	0.024
Female	1	(Reference)	–
BMI	1.02	0.94–1.1	0.724
Previous colonoscopy	0.79	0.60–1.03	0.786
Abdominal operation history	0.89	0.37–2.17	0.809
Diabetes mellitus	3.01	1.19–7.60	0.020
Educational level			
< Middle school	1	(Reference)	–
Middle school	1.7	0.11–26.14	0.703
High school	0.94	0.16–5.68	0.949
Graduate	1.75	0.68–4.52	0.250
Postgraduates	0.92	0.36–2.33	0.860
Annual income level, $			
< 10.000	1	(Reference)	–
10,000–20,000	0.71	0.06–7.81	0.777
> 20,000–30,000	0.97	0.23–4.06	0.963
> 30,000–40,000	0.45	0.17–1.18	0.103
> 40,000	0.81	0.39–1.67	0.572
Educational video			
Non-video group	3.06	1.73–5.42	<0.001
Video group	1	(Reference)	–
Multivariate analysis			
Sex			
Male	1.95	1.07–3.54	0.029
Female	1	(Reference)	
Diabetes mellitus	2.79	1.16–6.70	0.021
Educational video			
Non-video group	3.09	1.77–5.39	<0.001
Video group	1	(Reference)	–

### Polyp detection rate and procedure time

The polyp detection rate was not significantly different between groups. The rates were 19.0 % (48/252) in the non-video group and 19.2 % (48/250) in video group (*P* = 0.963). A significant difference in insertion time was observed between the control (6.1 ± 3.7 min) and experimental groups (5.5 ± 3.2 min; *P* = 0.043). However, no significant difference was found in withdrawal time (Table 4).

**Table 4:** Procedure time and polyp detection rate

	Non-video group	Video group	*p* value
Patients, *n*	252	250	
Insertion time, minutes (mean ± SD)	6.1 ± 3.7	5.5 ± 3.2	0.043
Withdrawal time, minutes (mean ± SD)	6.9 ± 3.9	6.6 ± 2.7	0.259
Patients with polyps, *n* (%)	34 (13.5)	34 (13.6)	0.963
Total number of polyps, *n* (%)	48 (19.0)	48 (19.2)	0.963

## Discussion

The defining characteristics of high-quality colonoscopy are the examination of the entire colon, optimal cleaning of the colon, and endoscopic withdrawal time of 6–10 min from cecum to rectum [15]. In this regard, many trials have been conducted to improve the quality of bowel preparation by patient education [9, 12, 14]. However, the effect of patient's education on bowel preparation has been limited thus far, with mixed findings. In one study, bowel preparation quality was superior among 205 patients receiving cartoon visual aids compared with those who received standard bowel preparation instructions. About 7 % of patients in the experimental group had poor preparation, compared with 18 % in the control group by using the Boston Bowel Preparation Scale (*P* = 0.02) [2]. On the other hand, a study of 969 patients found no impact on bowel preparation quality when compared between patients randomized to standard instructions versus instructions plus a visual aid, with a 91 % rate of adequate bowel preparation in the experimental group and 89 % adequate bowel preparation rate in the control group (*P* = 0.43) using the Boston Bowel Preparation Scale [12]. In the current study, the patients in the video group showed a greater reduction in Ottawa total score than the control groups (3.03 vs 4.21; *P* < 0.001). Furthermore, this educational video was effective to reduce the effort for cecal intubation by providing adequate bowel preparation. In the analysis of procedure times, the insertion time was significantly different between the control group and video groups (6.1 ± 3.7 vs 5.5 ± 3.2 min; *P* = 0.043).

The content of the educational video did not differ from the preparation manual received by all patients. However, the quality of preparations in the video group was significantly superior to the non-video group. This result means that conventional preparation manuals might have confused participants and failed to enhance patients’ bowel preparation. In addition, patients may forget key components of the bowel preparation process using the manual, because preparation instructions are often discussed in the outpatient clinic as early as 4 weeks before the procedure. The educational video could make the bowel preparation process more accessible to patient with simple words, illustrations, and video clips. Furthermore, providing a supplemental video convenient to access via the Internet at any time can be beneficial to increase compliance with bowel preparation.

The use of an educational video via the Internet has been increasingly used and objectively evaluated in other fields, and some investigators reported the benefit of the video clips as a platform for providing educational videos to the patients [16–18]. Therefore, we recommend the use of an Internet-based educational video with the standard bowel preparation manual. This could lead to achieve the improvement in patients’ understanding of the rationale for bowel preparation as well might enhance the quality of bowel preparation.

In previous studies on factors related to poor bowel preparation, male sex, diabetes mellitus, low education level, and low economic level were associated with poor bowel preparation [19, 20]. The results of our analysis are in line with some previous findings. In the multivariate analysis, males, people with diabetes mellitus, and those who did not use the educational video were significantly more likely to have poor bowel preparation. Nevertheless, education and income level did not result in any difference in quality of bowel preparation. However, our study was limited to the local enrolled population from Incheon, Korea, and its vicinity. The majority of patients underwent colonoscopy for health screening offered by some offices, and only 5.9 % patients (<20,000 US dollar) in current study were the low economic level judged by economic status in South Korea; thus, their education and economic level were not representative of the whole of society. Therefore, our result on education and economic level may contain selection bias. A future study focusing on education and economic levels would be required to properly understand associations between bowel preparation and these factors.

In our study, overall polyp detection rates were 19.1 % (96/502). This result was lower than those in previously published studies in which the polyp detection rate was >30 % [12, 14, 21]. We anticipated this result, since over half the subjects of our study had a previous colonoscopy history with polyp removal and were prone to have low polyp detection rate than naïve patients. In addition, the mean age of our subjects (48.24 ± 8.94 years) was younger than those of previous reports, in which the mean age were late fifties [12, 21]. Because polyp detection rate is increasing gradually by growing old, [22] the younger age would lead to low polyp detection rate of our study. The lack of detectable difference in ADRs between the two groups may be due to inadequate sample size, with a relatively young cohort who have a low incidence of polyps. We expected that the use of a larger general population may result in a significant difference in polyp detection rates, as the association between adequate bowel preparation and higher polyp detection rate is well known [23].

Our study has notable strengths. First, it is a well-designed, large-scale, colonoscopist-blinded, prospective, randomized trial. Second, we controlled for factors known to influence bowel preparation quality, such as the species of purgative agent, the timing of purgative administration, and the interval between bowel preparation and colonoscopy start time. However, our study has certain limitations. First, it was a community-based, single center study with enrolled patients representing the local residents of Incheon. Therefore, the participants might not represent the general population. In addition, the inter-endoscopist agreement for the Ottawa score was not validated in this study. Thus, there would be a risk of inter-observer bias in current study. Second, the effect of the educational video alone was not evaluated in current study. The educational video was used as a complementary preparation method in current study, so that further study would be needed to confirm the true effect of educational video alone for bowel preparation. Finally, our study only evaluated preparation quality with a single preparation method and did not compare other available preparation products and processes.

## Conclusion

In conclusion, an educational video might increase the quality of bowel preparation and decrease colonoscopists’ effort for cecal intubation. In addition, male sex and diabetes mellitus are significant risk factors for poor bowel preparation. Further studies with variable patient demographics and different preparation processes may be useful to evaluate the impact of an education video on bowel preparation.

### Abbreviations

OR, Odd ratio; Ottawa score, Ottawa Bowel Preparation Quality Scale.

## Additional file

Additional file 1: Video 1. Educational video for bowel preparation (MP4 47504 kb)
